# Surgical Treatment of Pulsatile Tinnitus Caused by the Sigmoid Sinus Diverticulum: A Preliminary Study

**DOI:** 10.1097/MD.0000000000000882

**Published:** 2015-05-29

**Authors:** Guo-Peng Wang, Rong Zeng, Xiao-Bo Ma, Zhao-Hui Liu, Zhen-Chang Wang, Shu-Sheng Gong

**Affiliations:** From the Department of Otolaryngology Head and Neck Surgery, Beijing Friendship Hospital, Capital Medical University, Beijing, China (G-PW, RZ, S-SG); Department of Otolaryngology Head and Neck Surgery, Beijing Tongren Hospital, Capital Medical University, Beijing, China (G-PW, RZ, X-BM, S-SG); Department of Radiology, Beijing Tongren Hospital, Capital Medical University, Beijing, China (Z-HL); and Department of Radiology, Beijing Friendship Hospital, Capital Medical University, Beijing, China (Z-CW).

## Abstract

The sigmoid sinus diverticulum (SSD) is an increasingly recognized cause of pulsatile tinnitus (PT). Surgical reconstruction of the sigmoid sinus wall has been found to be highly effective for SSD; however, surgical techniques still need to be refined to reduce the incidence of serious postoperative complications. Moreover, scrutinizing more cases of SSD is desirable for establishing the diagnostic criteria and standardizing the surgical technique. This study was retrospectively undertaken in 28 patients who were diagnosed with SSD upon computed tomography angiography (CTA) and digital subtraction angiography findings at Beijing Tongren Hospital in China. A majority of patients (20/28) presented with SSD and accompanying sigmoid sinus wall dehiscence (SSWD). Twenty-five patients opted to undergo sigmoid sinus wall reconstruction, and 3 patients refused surgery. Following surgery, 17 patients experienced complete resolution of PT, 3 patients experienced partial resolution, and 5 patients experienced no change in PT. No serious complications were found postoperatively. A comparative analysis of the preoperative and postoperative CTA findings suggested that completely resolving SSD and the accompanying SSWD resulted in the elimination of PT. In conclusion, SSD is generally accompanied by SSWD. Sigmoid sinus wall reconstruction is a safe and effective approach for the treatment of SSD. During surgery, completely resolving both SSD and SSWD is advisable, and maintaining the normal diameter of the sigmoid sinus is imperative.

## INTRODUCTION

Tinnitus, defined as the perception of an auditory sensation in the absence of an external stimulus,^1,2^ is a common disorder. Tinnitus is an annoying and disabling symptom in numerous individuals. Tinnitus may be classified as either nonpulsatile tinnitus or pulsatile tinnitus (PT). Nonpulsatile tinnitus, usually continuous and subjective, is markedly more common than PT. Because its pathogenesis is still unknown, etiological treatments for nonpulsatile tinnitus are limited. PT, whose rhythm usually coincides with the heartbeat, is relatively rare, and comprises approximately 4% of patients with tinnitus.^2,3^ PT is generally caused by vibrations from the turbulent blood flow of vascular structures that reach the cochlea.^1^ PT is surgically curable when the causative vascular anomalies or tumors are determined and eliminated.

Sigmoid sinus diverticulum (SSD) is a newly and increasingly recognized cause of PT. SSD is usually defined as a well-circumscribed sac in which the sigmoid sinus focally protrudes into the adjacent mastoid area.^4,5^ It was first reported as “a laterally placed sigmoid sinus”^6^ in 1995 and was also previously described as an “aneurysm of the sigmoid sinus.”^7–9^ SSD was previously considered an uncommon cause of PT; however, because an increasing number of PT patients with SSD have recently been reported, it has been established as the most common identifiable cause of venous-originating PT.^10–12^ Increasing attention is being paid to this structure.

Another explanation for the increased awareness of SSD by otologists and radiologists is that SSD is treatable with a high rate of success. Two approaches have been developed to treat PT patients with SSD successfully: endovascular coiling/stenting^7–9,13–15^ and transmastoid surgery (sigmoid sinus wall reconstruction).^4,16,17^ Endovascular treatment is used to embolize the diverticulum by coiling or stenting, thereby correcting the turbulent blood flow in SSD. However, there are some risks associated with endovascular treatment, such as coil migration, increased intracranial pressure, and thrombosis. To prevent thrombosis, anticoagulation is necessary during the perioperative period. Moreover, it fails to repair sigmoid sinus wall dehiscence (SSWD), which is also a significant cause of PT and co-occurs with SSD in many cases.^5,18,19^ In contrast, transmastoid surgery aims to excise the SSD and repair bony wall dehiscence using autologous or artificial materials. It involves few of the risks mentioned above because it does not require surgery on blood vessels, and no anticoagulation is necessary during the perioperative period. In addition, transmastoid surgery is curative for dehiscence alone, without diverticulum formation.^18,20^ Therefore, it is recommended as the preferred treatment for PT patients with SSD.^4,16^

Sigmoid sinus wall reconstruction was first reported by Otto et al in the successful treatment of 3 PT patients with SSD.^4^ In their study, following the skeletonization of the sigmoid sinus, the adjacent dura, and the diverticulum, the sigmoid sinus wall was reconstructed through the extraluminal placement of either the temporalis muscle and fascia or bone wax. Eisenman's group^16,17^ performed the surgery with a similar surgical technique, except that a soft-tissue graft of temporalis fascia or neuro-alloderm was interposed between the dura and the posterior fossa bony plate to reconstruct the soft tissue sinus wall. They found that although most patients (28/31) experienced complete resolution of PT after surgery, a few patients (3/31) failed to respond to the surgery, despite using identical surgical procedures.^17^ Two patients experienced serious postoperative complications: one with visual loss and intracranial hypertension and the other with progressive headache with ipsilateral sinus thrombosis.^16^ Increased intracranial pressure is also a risk when surgical reconstruction of the sigmoid sinus wall is undertaken for the treatment of PT from the prominent sigmoid sinus.^21^ Above all, transmastoid surgery is highly effective for PT caused by sigmoid sinus anomalies; however, it is inappropriate in a few cases, and vigilant monitoring for serious postoperative complications is essential. Furthermore, most studies of the treatment of SSD are case reports or small case series. Eisenman's group treated 13 SSD patients surgically, making it the largest case series on the surgical management of SSD.^17^ Many important aspects of SSD remain unknown, such as its pathogenesis, the diagnostic criteria, and the surgical techniques required. Therefore, experience with more cases of SSD is desirable to illuminate the aforementioned issues and to provide sufficient surgical refinement of sigmoid sinus wall reconstruction.

We previously reported on the clinical characteristics of sufferers of PT caused by SSD and wall dehiscence.^5,19^ Among them, 25 patients with SSD elected to undergo sigmoid sinus wall reconstruction. In this study, by comparative analysis of the patients’ preoperative and postoperative computed tomography angiography (CTA) findings, we presented our surgical outcomes of PT caused by SSD and summarized our surgical skills with the aims of improving the success rate of the surgery and reducing complications.

## METHODS

The study was retrospectively undertaken on patients hospitalized in the Department of Otolaryngology Head and Neck Surgery of Beijing Tongren Hospital between December 2008 and May 2012 with a chief complaint of PT. The diagnosis of SSD was made based on CTA and digital subtraction angiography (DSA) findings, which showed a well-circumscribed sac in which the sigmoid sinus was focally protruding into the adjacent mastoid air cells or cortex through a bone defect in the sigmoid sinus wall.^4,5,19,22^ The imaging methods used in CTA and DSA have been previously described.^5,19^ SSD patients with accompanying SSWD,^4^ in regions other than those involved in SSD, were also included in this study. However, patients with SSWD alone, without the formation of diverticula, and patients who were proven to have other definite causes of PT were excluded from this study.

Twenty-eight patients were diagnosed with SSD and recruited for this study, including 25 patients who opted to undergo surgical sigmoid sinus wall reconstruction (the operative group) and 3 patients who refused surgery (the nonoperative group). These 28 patients overlapped with the SSD patients in our previous studies^5,19^ in terms of a focus on their clinical characteristics and imaging features. No repeated data were presented in this study. The confidentiality of all patients was preserved, and each operative patient signed a written consent for the surgery. This study was conducted pursuant to the approval of the Committee on Medical Ethics of Beijing Tongren Hospital of China. The methods were carried out in accordance with the Declaration of Helsinki.

Preoperative data from the patients’ medical records were collected and assessed, including sex, age, medical history, physical examination, and radiological findings. Moreover, the severity of tinnitus and related distress was measured using the Tinnitus Handicap Inventory (THI) before surgery.

Surgical reconstruction of the sigmoid sinus wall was performed according to the previous literature^4^ with amendments aimed at reducing or excising the diverticulum and reconstructing the bone wall surrounding the sigmoid sinus. The surgical procedures in each case varied slightly. First, under general anaesthesia, the mastoid cortex was exposed after a postauricular incision and a pedicled myoperiosteal flap was made. During this process, the temporalis fascia was harvested. Next, the mastoid was opened, and autologous bone powders produced contemporaneously were collected. Then, the region surrounding the SSD, based on CTA and DSA findings, was skeletonized. Following extraluminal placement of the temporalis fascia and the autologous bone powders in sequence, the affected area was properly decompressed to a normal-appearing sinus wall. For those with accompanying SSWD, the defective wall was exposed and covered with temporalis fascia and bone powders. The graft was fixed with a medical adhesive (FAL, Beijing Fuaile Co., Beijing, China). Finally, the opened mastoid was covered with the pedicled myoperiosteal flap, and the skin was closed. After the surgery, a compressive mastoid dressing was placed.

All patients received regular follow-ups for at least 15 months by either telephone or return to the clinic. They were questioned for changes in PT. Based on the subjective report of symptoms, the outcomes of PT were classified into 3 levels: complete resolution, partial resolution, and no change. The patients’ conditions when follow-ups were also evaluated with the THI. In addition, postoperative complications were recorded for all patients. CTA and pure tone audiometry were performed only in patients who returned to the clinic.

Statistical analyses were performed using Student *t* tests (Prism 5, GraphPad Software, Inc., La Jolla, CA, USA) for the comparisons of age and body mass index (BMI) between the operative group and the nonoperative group. The statistical significance of differences was determined by ANOVA (Prism 5) to compare clinical features among the groups having different postoperative outcomes. Differences were considered to be significant when *P* ≤ 0.05, for all statistical tests.

## RESULTS

### Clinical Features of Patients

As shown in Table 1, the study population consisted of 28 patients, including 26 women and 2 men. All individuals were of Han Chinese nationality and had unilateral PT with a rhythm in synchrony with the heartbeat, including 10 left ears and 18 right ears. Preoperative CTA and DSA indicated that a majority of patients (20/28) presented with both SSD and SSWD. There were 25 patients who underwent surgery (the operative group) and 3 patients who refused surgery (the nonoperative group). The age of the patients suffering from PT in the operative group was 36.48 ± 9.19 years, which did not differ significantly from that of the nonoperative group (41.00 ± 17.69 years, *P* = 0.47). Similar results were observed when the average BMI was evaluated between the two groups (22.76 ± 2.68 vs 23.68 ± 1.34 kg/m^2^, *P* = 0.57).

**TABLE 1:**
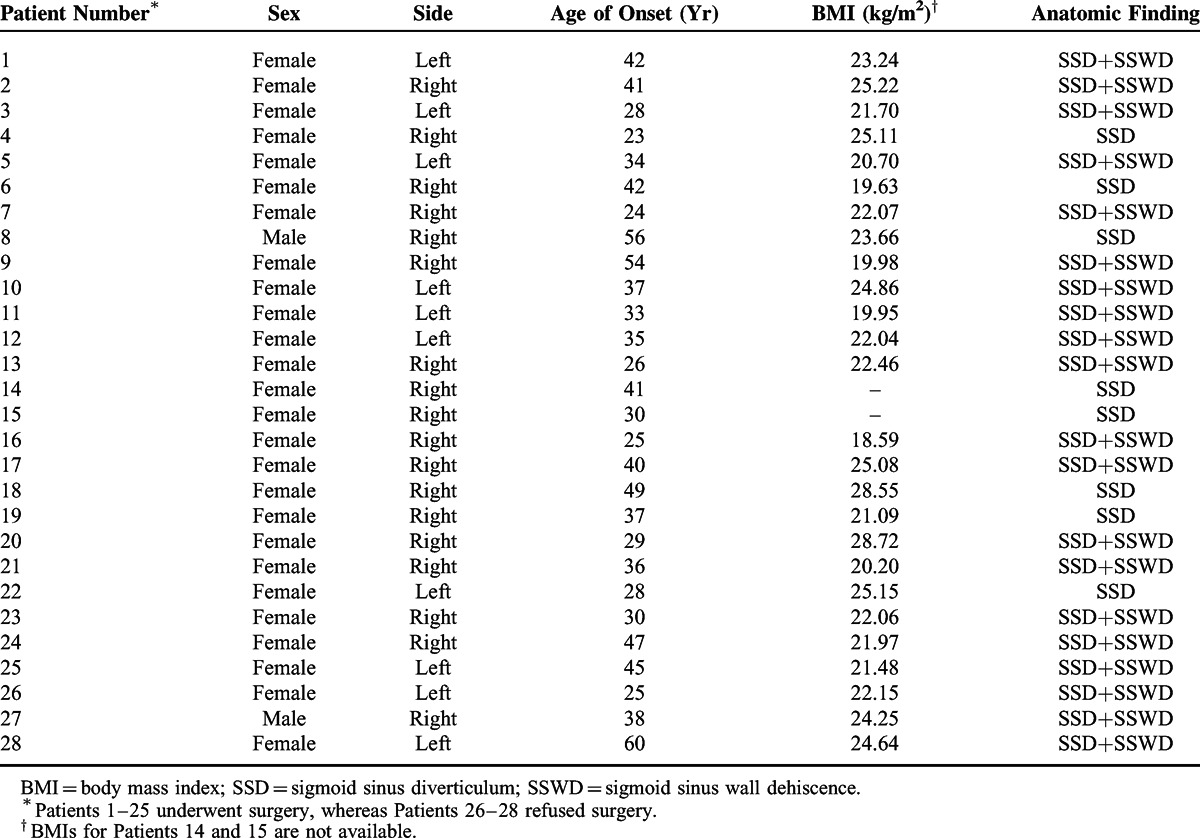
Preoperative Patient Information

### Intraoperative Findings

**FIGURE 1:**
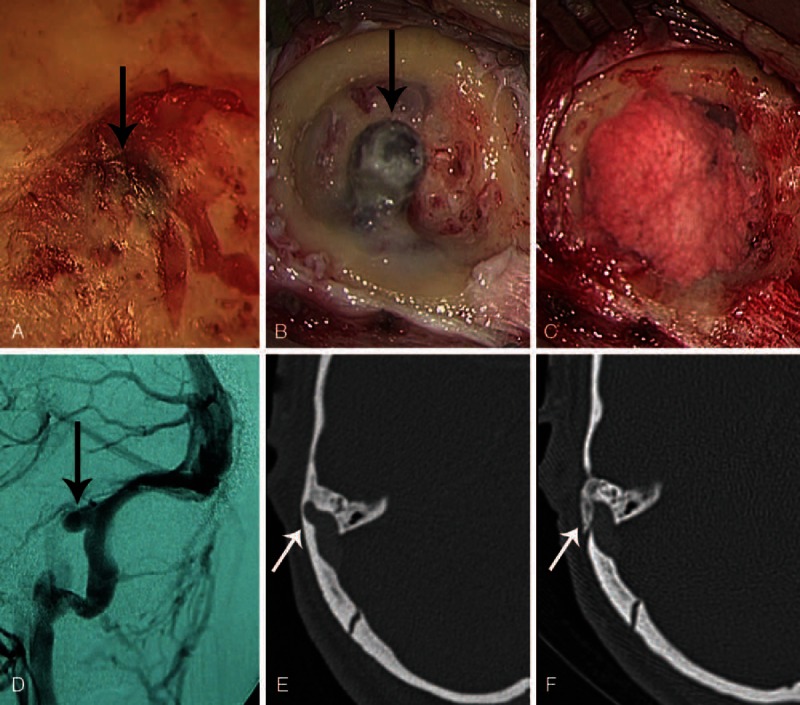
Intraoperative photographs (A–C) and preoperative (D and E) and postoperative (F) imaging findings for a patient (Patient 4) experiencing the complete resolution of PT following surgery. A, The diverticulum is shown through the thin, overlying mastoid cortex (arrow). B, The diverticulum is isolated following skeletonisation (arrow). C, The sigmoid sinus wall is reconstructed with fascia and autologous bone pate. D, Preoperative DSA image of the diverticulum (arrow). E, Preoperative CTA shows the diverticulum protruding into the adjacent mastoid region (arrow). F, Postoperative CTA, at the same level as in (E), demonstrates the elimination of the diverticulum, with a normal diameter of the sigmoid sinus (arrow).

The region of the diverticulum generally presented as a focal dark blue area through the thin, overlying mastoid cortex (Figure 1A). In a few cases, the diverticulum had even eroded through the mastoid cortex into subcutaneous tissues, which was evident once the mastoid cortex was exposed. The dura in the region of the diverticulum was generally fragile and could easily bleed. Following skeletonization (Figure 1B), the sigmoid sinus wall was repaired using temporalis fascia and autologous bone powders (Figure 1C), and the diverticulum was properly decompressed to a normal-appearing sinus wall. However, the sinus was never ligated or obliterated. Preoperative imaging findings of the patient presented in Figure 1A–C are shown in Figure 1D and E, and Figure 1F presents the postoperative CTA image showing that SSD has been eliminated. This patient (Patient 4) experienced complete resolution of PT after the surgery.

### Outcomes of PT and Postoperative Complications

All patients received regular follow-ups by either telephone or return to the clinic. Thirteen patients returned to the clinic for evaluation, whereas 15 patients followed up by telephone. The average follow-up time was 43.04 ± 15.74 months, ranging from 15 to 67 months. Outcomes of PT in the patients are shown in Table 2. In the operative group (Patients 1–25), following surgery, 17 patients experienced the complete resolution of PT (Group A), 3 patients experienced the partial resolution of PT (Group B), and 5 patients experienced no change in PT (Group C). Three patients (Patients 4, 14, and 19), who had experienced faint PT conditions only when engaged in heavy activities, were judged to have experienced complete resolution. The average THI value after surgery was 15.04 ± 21.86, with a difference value of 44.40 ± 28.25, compared with the preoperative THI value (59.44 ± 17.44). The nonoperative patients (Patients 26–28) continued to experience steady PT during follow-up. There was no obvious change in the initial and follow-up THI value (56.67 ± 9.02 vs 56.67 ± 10.26, respectively).

**TABLE 2:**
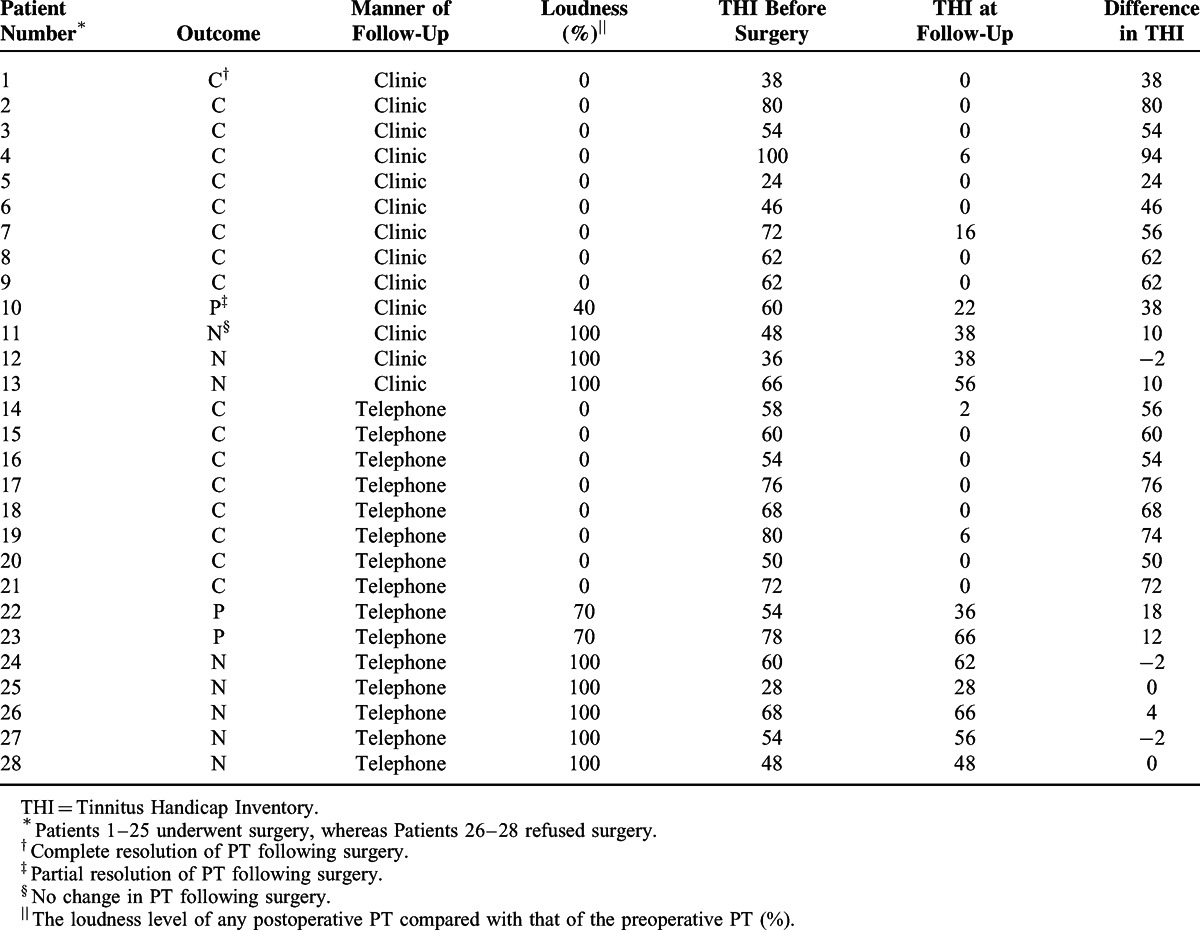
Outcomes of Pulsatile Tinnitus in the Patients

**FIGURE 2:**
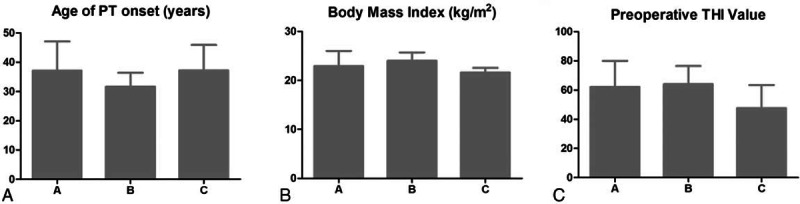
Comparison of clinical features among the groups. No significant differences (*P* > 0.05) are found with regard to age of pulsatile tinnitus (PT) onset (A), body mass index (B), or preoperative Tinnitus Handicap Inventory (THI) value (C). Groups A, B, and C represent the patients experiencing complete resolution of, partial resolution of, and no change in PT following surgery, respectively.

We compared clinical features among the patients in Groups A, B, and C. As shown in Figure 2, no significant differences were found for age of PT onset (*P* = 0.65), BMI (*P* = 0.45), or preoperative THI value (*P* = 0.24). In addition, 58.8% (10/17) of the patients in Group A presented with SSD accompanied by SSWD, and the rates in Group B and Group C were 66.7% (2/3) and 100% (5/5), respectively (Table 1).

After surgery, most cases had no complications. However, a few patients experienced persistent periauricular numbness, ear fullness, or collapse of the retroauricular area. One patient (Patient 7) achieved the complete resolution of PT, although she then suffered from a faint tinnitus of persistent rustling. Postoperative pure tone audiometry was performed in 12 patients, and there were no obvious changes compared with the preoperative data. No serious complications, such as headache, dizziness, or visual changes, were found.

### Comparative Analysis of the Preoperative and Postoperative CTA Findings

After surgery, 13 patients underwent CTA. Of these patients, 9 experienced complete resolution of their PT, 1 patient experienced partial resolution, and 3 reported no change in PT. The preoperative and postoperative CTA findings were compared in the 13 patients.

As shown in Table 1, 3 patients had SSD alone and 10 had both SSD and SSWD. In the 3 patients with SSD alone, the diverticula were resolved completely, as shown in Figure 1 for Patient 4, and all experienced complete resolution of PT postoperatively. Of the 10 patients with both SSD and SSWD, 1 had partial resolution of PT, 3 had no change in PT, and 6 had complete resolution of PT.

**FIGURE 3:**
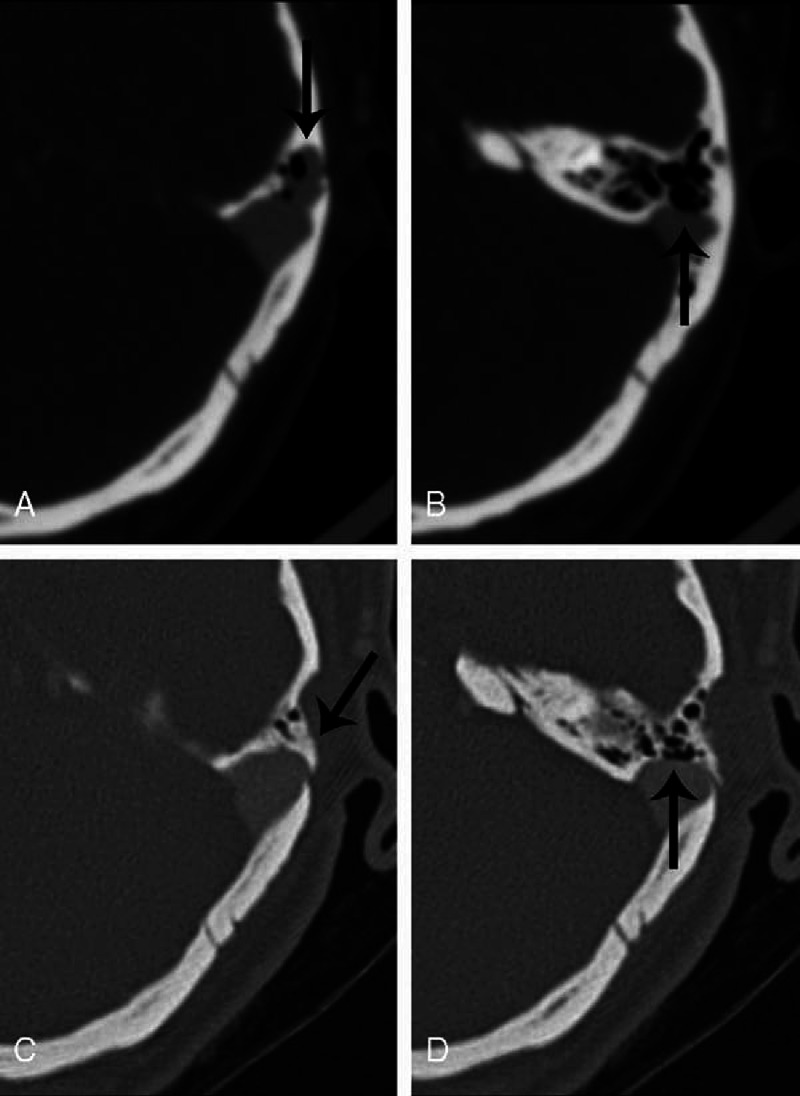
Preoperative (A and B) and postoperative (C and D) CTA images for a patient (Patient 10) experiencing the partial resolution of PT following surgery. A, The diverticulum erodes into the mastoid air cells (arrow). B, In the layer inferior to that of (A), the sigmoid sinus wall is dehiscent (arrow). C, The postoperative CTA image corresponding to the layer of (A) shows that the diverticulum is well eliminated (arrow). D, The postoperative CTA image corresponding to the layer of (B) shows that dehiscence of the wall remains (arrow).

**FIGURE 4:**
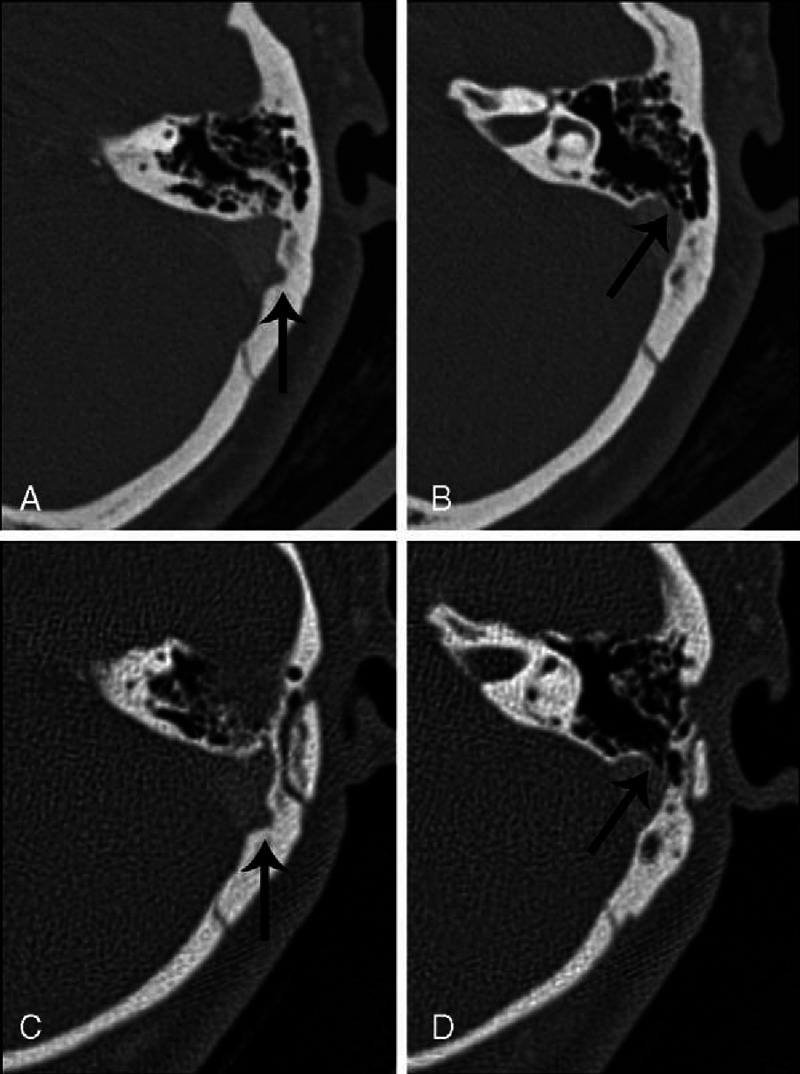
Preoperative (A and B) and postoperative (C and D) CTA images for a patient (Patient 12) whose PT was unchanged following surgery. A, The diverticulum erodes into the mastoid cortex (arrow). B, The sigmoid sinus wall is dehiscent, with an “air-on-sinus” sign in the inferior layer (arrow). C, The postoperative image corresponding to the layer of (A) shows that the diverticulum remains (arrow). D, The postoperative CTA image corresponding to the layer of (B) shows that the wall dehiscence remains (arrow).

**FIGURE 5:**
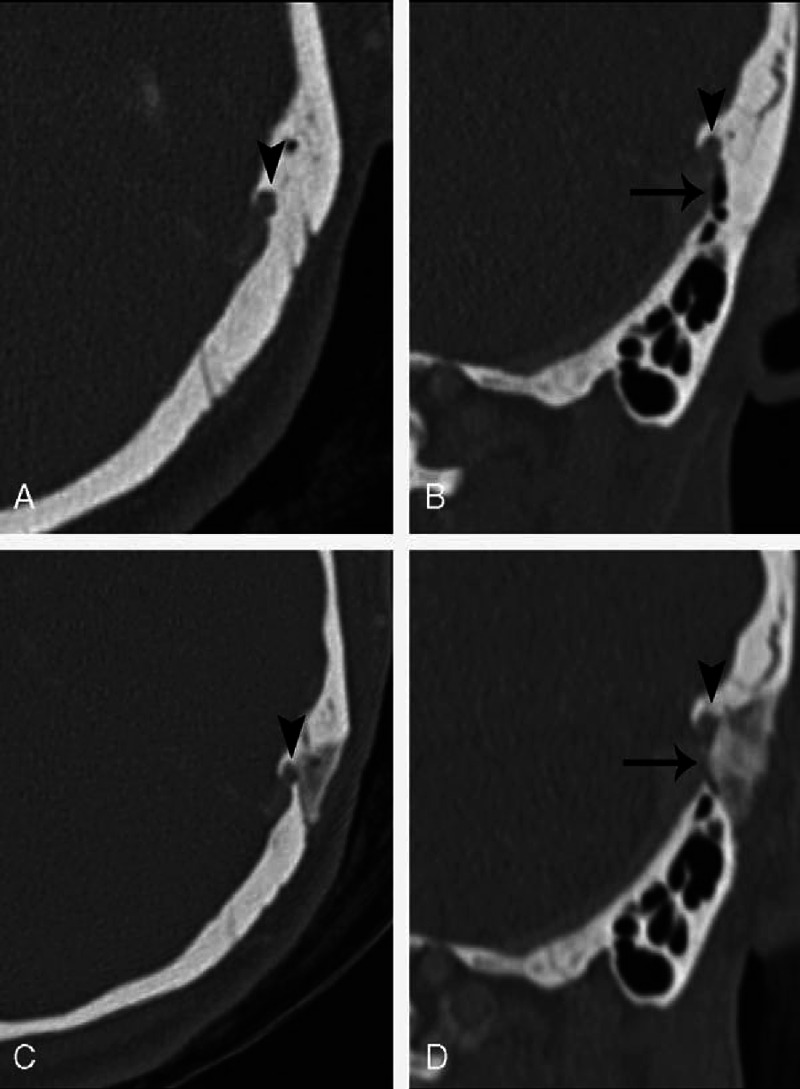
Preoperative (A and B) and postoperative (C and D) CTA images of a patient (Patient 5) experiencing the complete resolution of PT after surgery. With regard to approximate layers, (A) corresponds to (C), and (B) corresponds to (D). After surgery, the diverticulum (arrowheads) remains, but the dehiscent wall (arrows) is well reconstructed.

The CTA findings of the 10 patients with both SSD and SSWD are compared in Table 3. In the case with partial resolution of the PT (Patient 10), the diverticulum was successfully eliminated, but the dehiscent sigmoid sinus wall, whose layer was inferior to that of the diverticulum, was not repaired well (Figure 3). In each of the 3 cases experiencing no change in PT, both the SSD and dehiscence remained (Figure 4). In the 6 patients with complete resolution of PT, both anomalies were repaired completely in 1 case; the other 5 cases ultimately had well-repaired dehiscent sigmoid sinus walls, but had remaining (or incompletely eliminated) diverticula (Figure 5).

**TABLE 3:**
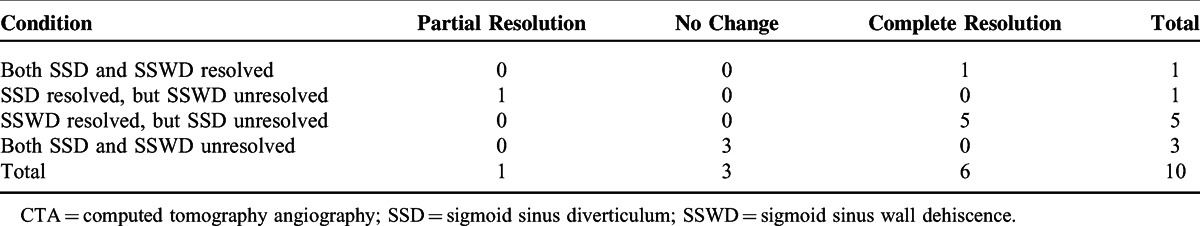
Comparative Analysis of CTA in 10 Patients with Both SSD and SSWD (Case Numbers)

## DISCUSSION

In the present study, 17 of 25 patients experienced the complete resolution of PT following surgery for sigmoid sinus wall reconstruction, and evident improvement in the THI values was detected between the preoperative and postoperative measurements. These findings suggest that the surgical treatment is highly effective for patients with PT caused by SSD. These data are consistent with previous reports.^4,16,17^

We also observed 3 patients (3/25) who experienced the partial resolution of PT and 5 patients (5/25) with no change in PT after surgery. No significant differences in age of PT onset, BMI, or preoperative THI value were found among Groups A, B, and C (Figure 2), indicating that these factors may be not contributors to the variance in the therapeutic effect. However, accompanying SSWD was more frequently observed in Groups B and C, especially in Group C (Table 1), compared with that in Group A. CTA findings for a patient experiencing partial resolution of PT (Table 3 and Figure 3) revealed that the SSWD was not well repaired, although the patient's SSD was successfully eliminated. Moreover, previous studies have shown that SSWD without SSD formation may also cause PT,^18,20^ suggesting that SSWD is another significant cause of PT apart from SSD. Above all, we speculate that the inadequate repair of the dehiscent sigmoid sinus wall might be one significant cause of the failure to achieve complete PT resolution. Nevertheless, more cases must still be collected to verify that assumption.

Although the pathogenesis of PT with SSD and SSWD is still unknown, there may be two major factors: turbulent blood flow in the region of the diverticulum and paucity of bone between the sigmoid sinus and middle ear. Thus, the surgery seeks to eliminate the audible turbulence in the diverticulum and to reconstruct the sigmoid sinus wall. For patients with SSD and SSWD, the dehiscent sigmoid sinus wall should be reconstructed simultaneously with the elimination of the diverticulum, which is supported by the complete resolution of PT in the patient who experienced comprehensive repair of both the SSD and SSWD.

In 3 cases who experienced no change in PT after the surgery, we found that the diverticula and dehiscence remained (Table 3 and Figure 4). Their surgeries were performed early in the study, when we first started performing the surgery. A lack of surgical experience and failure to expose the anomalies sufficiently appear to have resulted in unsuccessful treatment. Therefore, positioning the diverticulum and dehiscence based on preoperative imaging findings is imperative. In addition, because SSD may also exist in non-PT patients^5,11^ and because numerous PT cases may be idiopathic even after detailed physical examination and extensive radiological evaluation,^23^ other unknown factors may have caused PT in those patients experiencing surgical failure, although they underwent both CTA and DSA before the surgeries.

In most cases, we found that the dura in the region of the diverticulum was fragile and could easily bleed. Therefore, careful surgery is required, and greater caution should be taken when skeletonizing the diverticula. Bipolar cautery is advised for reducing the diverticula and retracting the sigmoid sinus adventitia from the surrounding bone.^16,20^ If bleeding results, covering the sigmoid sinus with a piece of temporalis fascia and depressing it for a while with a finger are advisable steps. And it is important not to lose packing materials within the lumen of the sinus.

Surprisingly, in 5 patients experiencing the complete resolution of PT, although the dehiscent sigmoid sinus wall was well repaired, the diverticulum remained or was not totally eliminated (Table 3 and Figure 5). In those patients, SSWD, but not SSD, may have been the major cause of PT. However, determining which factor serves as the major cause of PT in patients with both SSD and SSWD remains difficult. In addition, these results suggest that in patients with large diverticula having increased risk of bleeding, the complete elimination of the diverticulum is not required.

Tinnitus is a subjective symptom, with no objective clinical measure. The THI is widely endorsed clinically as a useful self-reporting assessment for quantifying the impact of tinnitus on daily life.^24,25^ In the present study, increases or decreases in the THI values were found in patients with no change in PT following surgery and in nonoperated patients, who continued to experience steady PT during follow-up (Table 2). Because the THI is a subjective questionnaire and the severity of tinnitus correlates with the individual's psychological response to the tinnitus, different intervals of THI values have been recommended for grading tinnitus severity.^26^ The fluctuation range of the THI values of patients with no change in PT was slight (<10); therefore, the change in the THI is approximately consistent with the patient's subjective report of PT.

Two severe postoperative complications of surgical sigmoid sinus wall reconstruction have been described in previous reports: increased intracranial hypertension and sinus thrombosis.^16,21^ In both studies, either a soft tissue graft or cortical bones had been inserted between the dura of the sigmoid sinus and the posterior fossa bony plate. Increased intracranial hypertension was relieved when a piece of bone was removed and the venous flow of the sigmoid sinus returned,^21^ demonstrating that this phenomenon is closely associated with excessive compression of the sigmoid sinus by bone insertion. In the present study, the affected area of SSD was properly decompressed to a normal-appearing sinus wall without the interposition of grafts, and none of the above complications was found. Therefore, maintaining the normal diameter of the sinus during surgery is important. Indeed, a few patients in our study experienced periauricular numbness, ear fullness, or collapse of the retroauricular area. Several cases were examined for acoustic impedance, and their results were normal (data not shown). These complications likely resulted from the postauricular incision and the absence of the partial mastoid cortex and air cells.

Several studies^11,17^ found that SSD manifested in patients with idiopathic intracranial hypertension (IIH). Both are frequently seen in middle-aged, obese females. In the current set of cases, a marked female preponderance was indeed found (Table 1); however, many cases had weights within a normal range, and no symptoms or signs of increased intracranial hypertension were found preoperatively. However, because neither lumbar puncture nor fundus examination was performed in our study, associations between SSD and IIH still cannot be excluded. To elucidate the precise relationship between these two entities, it would be essential to design a prospective study in which neurologic and ophthalmologic evaluations are performed in patients with SSD and in which CTA, focusing on the temporal bone, is undertaken in patients with IIH.

Limitations of this report include the retrospective nature of the study, being based on the experiences of a single center. Our study recruited 28 SSD patients, of whom 25 opted to undergo sigmoid sinus wall reconstruction. To our knowledge, this makes it the largest number of cases focusing on the surgical treatment of SSD, although the number of recruited patients was small. The clinical features of PT patients with SSD were assessed, and preoperative and postoperative CTA were comparatively analyzed, which may provide insights into the diagnosis and management of SSD in the future.

## CONCLUSION

SSD is a significant cause of PT. It generally co-occurs with SSWD. Transmastoid sigmoid sinus wall reconstruction is a highly safe and effective approach for the treatment of SSD. During surgery, the complete resolution of the diverticulum and dehiscence is advisable, and maintaining the normal diameter of the sigmoid sinus is imperative.
